# The ‘false hope’ argument in discussions on expanded access to investigational drugs: a critical assessment

**DOI:** 10.1007/s11019-022-10106-y

**Published:** 2022-08-11

**Authors:** Marjolijn Hordijk, Stefan F. Vermeulen, Eline M. Bunnik

**Affiliations:** Department of Medical Ethics, Philosophy and History of Medicine, Erasmus MC, University Medical Centre Rotterdam, Wytemaweg 80, 3015 Rotterdam, CN The Netherlands

**Keywords:** Expanded access, Compassionate use, Investigational drugs, Drug registration, False hope, Ethics.

## Abstract

When seriously ill patients reach the end of the standard treatment trajectory for their condition, they may qualify for the use of unapproved, investigational drugs regulated via expanded access programs. In medical-ethical discourse, it is often argued that expanded access to investigational drugs raises ‘false hope’ among patients and is therefore undesirable. We set out to investigate what is meant by the false hope argument in this discourse. In this paper, we identify and analyze five versions of the false hope argument which we call: (1) the limited chance at benefit argument, (2) the side effects outweighing benefits argument, (3) the opportunity costs argument, (4) the impossibility of making informed decisions argument, and (5) the difficulty of gaining access argument. We argue that the majority of these five versions do not provide normative ground for disqualifying patients’ hopes as false. Only when hope is rooted in a mistaken belief, for example, about the likelihood of benefits or chances on medical risks, or when hope is directed at something that cannot possibly be obtained, should it be considered false. If patients are adequately informed about their odds of obtaining medical benefit, however small, and about the risks associated with an investigational treatment, it is unjustified to consider patients’ hopes to be false, and hence, to deny them access to investigational drug based on that argument.

ORCID S.F. Vermeulen: 0000-0002-8797-9172.

ORCID E.M. Bunnik: 0000-0003-1481-6222.

**Statements and Declarations**.

## Competing interests:

The authors have no competing interests to declare that are relevant to the content of this article.

**The ‘false hope’ argument in discussions on expanded access to investigational drugs: a critical assessment**.

## Introduction

Patients who cannot, or can no longer, be treated using standard treatment regimens may be confronted with severe disability, or pending death. Under certain conditions, patients who find themselves in such back-against-the-wall situations may qualify for expanded access to investigational drugs. Expanded access aims to offer seriously or terminally ill patients access to unapproved, investigational drugs outside of clinical trials. Although regulations differ internationally, expanded access can generally be granted only if patients satisfy strict eligibility criteria: patients must suffer from seriously or life-threatening illness, must have no suitable approved alternatives left and must not be eligible for enrolment in clinical trials (European Commission 2004; US Food and Drug Administration 2020). Expanded access can be requested for individual patients by treating physicians or can be granted through industry-initiated programs, usually referred to as named-patient or compassionate use programs, respectively.

Since its inception, expanded access to investigational drugs has raised ethical discussions. Recurrent topics in these discussions are concerns about the safety and efficacy of investigational drugs (Darrow et al. 2015; Gesme 2007; Wieseler et al. 2019) together with the lack of clinical data on investigational drugs (Pearlman 2018), funding issues (Bunnik et al. 2017), and difficulties of actually obtaining investigational drugs in practice (Gesme 2007, Pearlman 2021). A frequently recurring concern in these discussions is ‘false hope’ (Rubin 2015; Zuckerman 2017; The Lancet 2018; Cohen 2018; Jaggar 2018): the concern that patients’ hopes of profiting from investigational treatments are ill-founded, for instance because of the potentially limited chances of medical benefit (Walker 2014) or the risks of side effects (Carter-Johnson 2015) associated with these treatments. False hope is often used as an argument against expanded access to investigational treatments. The Argument of ‘False Hope’ (hereafter referred to as AoFH) is not unique to the field of expanded access. It has also been used in oncology (Chamberlain and Sullivan 2019), consumer medicine (Eijkholt 2020), and more recently in off-label drugs for COVID-19 (Washington Post 2020). The AoFH is not a single, uniform argument but comes in versions built upon different underlying rationales for considering patients’ hopes false. Often, these rationales are not made explicit. This renders it difficult to determine whether the AoFH serves as a valid argument in discussions on expanded access to investigational medical treatments. Also, it may be perceived as patronizing by some patients, as it suggests that patients do not understand their chances at medical benefit, and can be easily misled (Warren & Junod 2014).

This article aims to demarcate and critically assess the ways in which the AoFH is used in the discussions on expanded access to investigational drugs. In doing so, it aims firstly to gain a better understanding of what false hope is taken to mean within the expanded access discussions, and secondly to assess to what extent false hope has a role to play as an argument against expanded access to investigational drugs.

It should be noted that any such assessment may be complicated by the complex nature of the concept of (false) hope. In the literature, the concept of (false) hope has been the subject of extensive analysis in varying disciplines, such as medicine and healthcare sciences (Coughlin 2006, Schrank et al. 2008), philosophy (Day 1969; Musschenga 2019), theology (Moltmann 1968) and psychology (Snyder 2002; Feldman et al. 2009), yet a singular definition of (false) hope seems to be lacking. Across disciplines, hope has been defined in varying ways that emphasize different aspects or ‘modes’ of hope, which may be elicited in different contexts (Webb 2007). For example, hope has been described as primarily emotion-based (Bruininks and Malle 2005; Stengers 2002; Scioli et al. 2011), as a feeling of joy brought about by anticipating something that is wished for (Spinoza 1985). In context of disease, hope is also considered a therapeutic end in itself – as something that should be aimed for because it may help improve a patient’s life, alleviate their symptoms, and serve as a resource to cope with life’s challenges (McClement & Chochinov 2008). Others have stressed the rational aspect of hope (e.g. Snyder 2000a), defining it, for instance, as a longing for something that is linked with an explicit probability-belief (Day 1969, Hume 1896). Pleeging et al. (2021) also propose such a rational conception of hope, which is consistent with most interpretations of hope across these disciplines. In this dominant conception, hope consists *at least* in (a) a desire for something (the *conative* aspect of hope), coupled with (b) a belief in a possible, though uncertain goal (the *cognitive* aspect of hope) (Musschenga 2019). While this conception may not capture all aspects of hope, we will use it in our analysis.

In this article, we do not wish to add to the general ethico-philosophical discussion on the concept of (false) hope. We are solely concerned with finding ourselves a better understanding of what false hope is taken to mean in context of expanded access to investigational treatments, notably with decomposing the AoFH’s underlying rationales for considering hope false. We searched for the AoFH in discussions of expanded access to investigational medical treatments in ethical, professional and layman publications, including scientific publications in PubMed, medical-ethical literature, newspapers, websites of patient associations and other public sources. We then catalogued the five main versions of the AoFH that occurred most prominently in these publications, and named these versions respectively: (I) the limited chance at benefit argument, (II) the side effects outweighing benefits argument, (III) the opportunity costs argument, (IV) the mistaken belief argument, and (V) the difficulty of gaining access argument. Below, we will present these five versions of the AoFH, analyze the rationales on which they are built, and assess whether these rationales are justified. Also, we discuss whether they serve as valid arguments against the provision of expanded access to investigational medical treatments.

**Five main versions of the AoFH**.

### I) the limited chance at benefit argument

A first version of the AoFH contends that the hope raised in patients by offering them expanded access to investigational drugs is false due to the limited chance of medical benefit. Thus, if patients pursue investigational drugs in order to obtain medical benefit, their hopes are false because they are in fact unlikely to gain actual medical benefit from such drugs. For example, it has been mentioned that access to investigational drugs “will give dying patients false hope since only 10% of drugs are deemed worth the risk and make it through clinical trials” (Walker 2014).

It is true that approximately 90% of the drugs entering phase I clinical trials fail to gain marketing approval (Hay et al. 2014). In early-phase clinical trials, the average response rate (the percentage of patients in which an investigational treatment has a beneficial effect) is very low. Meta-analyses show objective response rates (ORRs) in the neighborhood of 5% and fatal toxicities in approximately 0.5% of patients (Roberts et al. 2004; Horstmann et al. 2005; Italiano et al. 2008). Proponents argue however that these response rates are consistent with those for several US Food and Drug Administration (FDA) approved anticancer drugs (Weber 2015) , Agrawal 2003). Furthermore, expanded excess to investigational drugs is not limited to early-phase trials. Rather, it almost exclusively concerns investigational drugs in late-phase II, or even post-phase III trials, as in many countries, regulations allow for expanded access no earlier than late phase II (Puthumana et al. 2018). With phase-progression, the chances at medical benefit will likely increase. Literature shows that, as opposed to the often fairly limited success rates of early phase trials, over 60% of the drugs tested in phase III trials do eventually advance to marketing approval (DiMasi et al. 2003). The percentage of drugs offered through expanded access programs registered on ClinicalTrials.gov reaching marketing approval in the US (76%) is even higher (Miller et al. 2017). Most investigational drugs offered through expanded access programs, thus, eventually reach marketing authorization, and the chances at benefit obtainable from investigational drugs need not be small.

But even if it is assumed that the chances of benefit offered by an investigational treatment available through expanded access *are* small, and that patients indeed pursue expanded access because they hope to obtain medical benefit – a patient’s hope need not be false. In order to explain this, it may be helpful to examine more closely the *cognitive* aspect of hope, as stemming from the dominant conception of hope. The cognitive aspect of hope consists in a true belief that that which is desired, whatever that may be, *can* be obtained or has the potential to come about (Musschenga 2019). Hope, then, may be considered false when it is built upon false belief regarding the potential of that which is desired for coming about. The limited chance at benefit argument seems to imply the latter: hope is deemed false because it compromises the cognitive aspect of hope. This may be either because patients have mistaken beliefs about the likelihood of obtaining what is desired, or because they accept a chance of benefit that is deemed too small to accept.

For hope to be valid (not false), patients should naturally be adequately informed about the nature of the investigational treatment. An adequate informed consent process is required in most countries’ regulations for expanded access. Within the informed consent process for expanded access, which should be more rigorous than that for standard medical procedures, it is up to the treating physician to verify and guarantee that patients understand the nature of their treatment, the risks and potential benefits, and the special status of expanded access to investigational drugs (US Food and Drug Administration 2016), and to correct any misunderstandings or mistaken beliefs. In that way, the risk that patients decide upon something that they do not understand, is minimized. If despite being offered relevant information, a patient’s hope to gain benefit from expanded access is still based on mistaken beliefs, then his or her hope may indeed be considered false.

In relation to the second reason for considering the hope raised in expanded access false, it may be pointed out that this reason presupposes some likelihood-threshold below which people should not hope in the first place. But this does not follow from the cognitive aspect of hope, as this requires only that that which is desired is not *wholly* beyond reach. Thus, only when it is absolutely impossible to obtain benefit from some investigational drug, hope may be deemed false, at least from a cognitive perspective. At the same time however, people may have different hope-thresholds (Musschenga 2019). For some, a 10% chance at benefit may seem a reasonable basis for having hope for a medical treatment, while for others, a 40% chance at benefit may not suffice. One’s hope-threshold may depend on contextual factors, such as the medical risks and burdens of the treatment or the availability of alternatives, but may also be person- and value-dependent (Webb 2007; Stevens et al. 2014). Because the thresholds of hope differ between individuals, hope can seem to be justified in the eyes of one individual, but less so, or not so, in the eyes of someone else. Yet, seen from the cognitive aspect of hope, everything above zero counts, and hope in the context of small chances at benefit need not be false as long as the person(s) involved have true beliefs about these (small) chances.

### II) the side effects outweighing benefits argument

A second version of AoFH concerns the likelihood of negative side effects associated with investigational drugs, and therefore the risk of inflicting of harm to patients. As argued in literature: “a patient is far more likely to receive a drug candidate that will either do nothing to help the condition and/or have significant adverse side effects beyond the symptoms caused by the disease. Thus, patients who ask for drug candidates that have merely completed a Phase 1 trial very likely are banking on a false hope and may even have their lifespan decreased or their quality of life diminished” (Carter-Johnson 2015).

This version of the AoFH entails that a patient’s hope to benefit from an investigational drug is false because she fails to acknowledge the risk of negative side effects. Whilst hoping for benefit, chances are there that the drug will lead to harm rather than benefits, and hence to the opposite of what is hoped for.

As stressed previously, it should be noted that expanded access does not usually concern early-phase clinical trials, as the example above may unjustifiably suggest. Nonetheless, it is true that investigational drugs *can* have negative side effects, and can even cause serious adverse events. As said, given the lack of safety data, as these drugs are still being evaluated in clinical trials, side effects may not (yet) be known. A historical example may be found in immune checkpoint inhibitors, a group of oncological therapies that have now been approved for the treatment of many types of malignant disease (Bagchi et al. 2021). Although immune checkpoint inhibitors are effective, they are associated with severe, even fatal side effects, including cardiac events (Wang 2018), and severe immune-related adverse events such as pancytopenia or aplastic anemia (Delanoy 2019), in a minority of patients. At the time of early-phase clinical trials of immune checkpoint inhibitors, these risks may not have yet been known. Patients may have tried these therapies via expanded access programs without having been aware of these risks, and may have suffered the consequences. Yet the question we need to answer is whether in light of (significant) chances of (serious) negative side effects, a patient’s hope to benefit from the treatment should be considered false.

This question can be answered along the lines of our response to the limited chance at benefit argument. For this argument again implies a problem with the cognitive aspect of hope: the likelihood of obtaining what is desired (medical benefit) is overshadowed by a greater likelihood of gaining precisely the contrary of what is hoped for (harm). For reasons laid out above, we shall assume that patients pursuing expanded access are well-informed about their chances at benefit as well as about the risks associated with the investigational treatment. Here again, it may be argued that, from a cognitive perspective, hope in light of an unfavorable risk-benefit ratio should not be considered false – because all that hope in this respect requires is that that which is hoped for is not entirely beyond reach, irrespective of the associated risks.

The argument of the relativity of hope-thresholds may be iterated here, as well. In this case, it may be stressed that patients who qualify for expanded access have run out of standard treatment options and are faced with serious or life-threatening illness. For them, the risk-benefit ratio of an investigational treatment may seem favorable more easily than for others. When irrevocably facing death or serious impairment, with their backs against the wall, patients may lower their thresholds and adapt their evaluations of the risks of side effects simply because they have little left to lose: “after all, in facing death, what safety risk can there be that could possibly be worse?” (Caplan 2007). This feeds, however, into a further argument that will be discussed below, namely: the argument that patients who are suffering from serious and life-threatening disease do have a lot to lose, and must be protected against being exposed to the risks and burdens associated with trying unapproved medical treatments. Of course, it is not at all the case that patients with serious and life-threatening diseases have nothing to lose. On the contrary, they may have more to lose than others.

#### III) the opportunity costs argument

This version of AoFH focusses on the opportunities that patients who qualify for expanded access to investigational drugs – patients suffering from serious or life-threatening illness – potentially miss out on when pursuing investigational treatments. These opportunities include adequate palliative care and a psychologically and socially peaceful end of life. In the literature it is argued for example that the “allure of promising new drugs continues to engender false hope, which has all too often diverted time, resources, and attention from more appropriate efforts to minimize symptoms and enhance the quality of life for terminally ill patients and their families” (Gesme 2007).

This version of AoFH suggests that patients who are facing life-threatening illnesses and have exhausted standard treatment options hope to gain benefit from investigational drugs is false because more benefit may be gained, or at least patients may be better off, with high-quality palliative care and acceptance of their pending death. Pursuing expanded access would only direct their attention away from these more valuable options. In this case, the primary question becomes whether hoping to gain benefit from expanded access while there are other, more beneficial options available, renders hope false.

In providing an answer to this question it may be useful to refer to the second aspect of the dominant conception of hope as described in literature: the *conative* aspect of hope. This aspect entails the idea that hope consists in a desire for something or a longing for something (the *object* of hope) to come about (Musschenga 2019). When interpreted in light of the conative aspect of hope, the opportunity costs argument implies that the hope raised by the prospect of expanded access to investigational drugs is false because it turns patients’ desires towards the wrong object, i.e. towards something that should not be an object of hope at all, because there are more desirable alternatives at hand.

Support for this claim may partly be found in the great value that is rightly attributed to adequate palliative healthcare. Treating physicians may quite understandably believe that in the latest phases of their patients’ lives, patients are better off being treated symptomatically. Medical treatment is then directed at the provision of comfort care, the maintaining of quality of life, and the minimizing of suffering (National coalition HPC 2018, WHO 2020). Pursuing expanded access may cause patients to attribute their hopes towards the ‘wrong’ object (i.e., an investigational drug), and keep them from striving for arguably more important or more beneficial goals, such as acceptance of death and saying goodbyes (i.e. a better end of life).

Importantly, however, expanded access to investigational drugs need not to stand in the way of palliative care. After expanded access has been tried, and in the case the treatment proves unsuccessful, palliative care can be initiated or intensified. Here we argue in favor of respect for autonomy. When and only if people are ready, it can be beneficial to start on a trajectory of providing end-of-life care. When people are not ready, when they do not want to accept end-of-life care, and prefer to pursue relevant opportunities for expanded access, people are probably not better off by being denied these opportunities, even if these are associated with only small chances at benefit. We acknowledge the value of palliative care for individual patients, and we argue that, as explained before, as long as patients are adequately informed about available palliative care options, their hope to nonetheless obtain (benefit from) investigational treatments cannot be said to be false.

It may as well be noted that what a good end of life entails cannot be established generically, but is both subjective and dynamic, varying between patients as well as over a patient’s lifetime, depending on factors such as age, cultural group, religious background and disease phase (Krikorian et al. 2020). Although literature suggests that factors indicated by patients as facilitating a good death are to some extent similar, and include pain control, gaining life-closure, and clear decision-making (Krikorian et al. 2020), the weighing of the importance of these factors, also against other factors, may be subjective. And not only do patients’ ways of confronting and coping with death differ (McKechnie 2007, Wright 2003), but also do the ways differ in which people achieve (those factors associated with) a good death (McKechnie 2007). For example, some terminally ill patients may perhaps not reach closure by accepting death or saying goodbyes. Literature suggests as well that terminally ill people attribute great importance to their health (Campbell 1999). To some, optimization of their (chances at better) health may be valued more highly than saying goodbyes, and they may therefore be more inclined to pursue further treatments that may be beneficial to their health. Due to such interpersonal differences, it could be that a treating physician feels that palliative options might be more favorable, while a well-informed seriously ill patient reasonably concludes otherwise. The principle of respect for autonomy implies that the patient’s conclusion should be acknowledged.

### IV) the mistaken belief argument

A fourth version of the AoFH involves the suggestion that seriously ill patients who have run out of approved treatment options might make an over-estimation of the potential benefits and/or an under-estimation of the associated risks, of the unapproved treatment. Consequently, they fail to make adequately informed (and thus non-autonomous) decisions (Darrow et al. 2015; Caplan 2007) about expanded access. This argument again suggests that hope is false because the cognitive aspect of hope is compromised, as it is based on a misunderstanding of relevant facts. Thus, the argument suggests, informed consent is not achieved.

In medical ethics, informed consent is traditionally defined by Faden and Beauchamp (1986) as an autonomous authorization by a patient for a professional to carry out some medical trajectory. Informed consent requires (1) substantial understanding, (2) in the absence of control by others, and (3) intentionality. Thus, the informed consent requirement, which, as said, is often also a legal prerequisite for granting patients access to investigational treatments (US Food and Drug Administration 2016), should ideally guarantee that patients are adequately informed and decide autonomously, based on adequate information, and free from pressure or coercion, whether they will undergo an investigational treatment.

To date, little is known about patient decision-making in the context of expanded access. Yet for patients who qualify for expanded access, making informed treatment decisions may indeed be difficult. Literature suggests that decision-making may be more easily impaired when patients are seriously ill (Kolva et al. 2018, Sorger et al. 2017, Burton et al. 2012), as their capacity for reasoning and understanding may decrease (Kolva et al. 2018). This may not only be due to their somatic condition, but also to the mental, social and spiritual effects of serious disease (Wilson et al. 2016; Kelley and Morrison 2015). Making informed decisions is likely to be even harder when unapproved treatments are concerned, as less evidence is available about relevant facts, such as safety, efficacy, and side effects. Especially in back-against-the-wall situations, patients may perhaps be less capable of making cool-headed estimations of their chances at benefit and may be more prone to overly optimistic expectations that do not correlate with the (limited) available evidence, as, for instance, fear or despair may become more pronounced. Also, suffering from life-threatening illness – especially in the absence of available alternatives – may exert some coercive influence, which may render patients less free to make autonomous decisions. If patients indeed feel pressured into trying investigational treatments, or if they fail to make adequate estimations of the harm-benefit ratios of investigational treatments, then they are not adequately informed, and, in this sense, their hopes may be considered false. In this case, again, the cognitive aspect of hope is compromised. For hope is then rooted either in a decision that is made unfreely, that is not based on (true) belief at all, or in a false belief about the degree to which the attainment of what is desired and the avoidance of what is not desired, is possible.

Whereas the above suggests that patients who qualify for expanded access may experience difficulties in making adequate assessments of their chances at benefit, this need notably not be true of all those patients. What it *does* suggest is that there is a pressing need for adequate informed consent processes in expanded access.

### V) the difficulty of gaining access to investigational drugs argument

The fifth version of the AoFH concerns the practical feasibility of gaining access to investigational treatments through expanded access programs. For expanded access to succeed, a patient must decide to want to try an investigational drug, a treating physician must be willing to prescribe it, a hospital pharmacist must be able to assist with making the request and arranging the logistics, health authorities and/or research ethics review committees or institutional review boards must evaluate and approve the request, the pharmaceutical company must be willing and able to supply the drug, and somehow the costs of the process must be covered (Bunnik et al. 2017). Patients who start on a road towards expanded access can eventually be denied the treatment if one or more of these actors withhold their approval or cooperation, and it may turn out unfeasible to obtain expanded access in practice.

Indeed, it is true that in many countries, there are obstacles that may hinder expanded access to investigational drugs in practice, meaning that after attempts of a treating physician to gain access, patients may not receive the drug. Examples of these obstacles are reimbursement issues, lack of familiarity with and the time-consuming nature of the application procedure, and lack of institutional support (Moerdler et al. 2019; Bunnik and Aarts 2021; Vermeulen et al. 2021).

Here again it may be useful to resort to the cognitive aspect of hope. We have already seen that the cognitive aspect of hope requires only that that what is desired is not *wholly* beyond reach. Hope is not false when the chances of success are small, or even very small. In fact, *uncertainty* is what hope is all about. It makes little sense to hope for something when one is (near) certain that this something will be obtained. Perhaps we could even sensibly argue that one is more prone to hope when one’s chances are small, since when one is near-certain that something will be acquired, one need not hope for it, but simply assume that one will likely obtain that which is desired. The cognitive aspect of hope requires only that the chances of obtaining the drug are not zero. So, if there is a chance that a patient may obtain a drug, hope should not be deemed false. This means that only if expanded access were prohibited or if it were absolutely certain that access was impossible (e.g., due to hospital regulations, local safety requirements, or company policies), hope may be deemed false. In most cases, however, although it may be difficult to actually gain access to investigational treatments, especially in some healthcare systems (Vermeulen et al. 2021), the chances of gaining access are not zero, and hope is therefore not false.

#### Concluding remarks

In this article, we have analyzed the five most prominent versions of the Argument of False Hope (AoFH) as used in bioethical discussions as a means to argue against expanded access to unapproved, investigational treatments. We have investigated the underlying rationales implicit in these versions of the AoFH, and argued that most of them do not provide normative ground for disqualifying patients’ hopes as false. Only when hope is rooted in mistaken beliefs, for instance, about the likelihood of benefits or the medical risks, or when hope is directed at something that cannot possibly be obtained, should it be considered false. Thus, if patients are adequately informed about their odds of obtaining medical benefit (however small) and about the risks associated with an investigational treatment, it is unjustified to consider patients’ hopes to be false, and hence, to deny them access to investigational drug based on that argument.

Despite their ill-founded rationales, all five versions of the AoFH are nonetheless widespread in scholarly and public discussions on expanded access to investigational drugs. They may therefore have a substantial impact on practices and uptake of expanded access. For instance, patients may be withheld information about opportunities to access investigational treatments with an appeal to the AoFH. This is problematic especially when the AoFH is built on an unsound rationale. We therefore suggest that, if the AoFH is brought to the fore in discussions on expanded access, the underlying rationale for considering patients’ hope to be false, should be made explicit. It is likely that in many cases, the perceived falseness of hope may be reducible to differences in hope thresholds or differences in valuations of the (likelihood of) benefits or side effects for patients, thereby rendering hope not false, but merely differently attributed.

This is not to say that expanded access does not raise ethical concerns, or that medical professionals should always act in accordance with patients’ desires to pursue expanded access to investigational drugs without giving extra thought to the balance of risks and benefits associated with expanded access in light of available alternatives, including palliative care options. On the contrary, given the high degree of uncertainty involved, deciding whether to pursue expanded access programs is obviously complex. As expanded access is a last resort for patients in back-against-the-wall situations, doctors should be aware that hope thresholds may differ between persons, and that consequently, patients and physicians may have different ideas about whether and to what extent investigational treatments are worthy of pursuit. The medical community should however proceed carefully and not automatically dismiss patients’ hopes as false. As an *ultimum remedium*, expanded access should always be given extra careful consideration in discussions between patients and medical professionals, and adequate informed consent – including the correction of false beliefs – remains an important precondition.

Further research should consider the informed consent process for expanded access to investigational drugs both empirically, from physicians’ and patients’ perspectives, and normatively. This should help determine what patients need to know about expanded access before asking their informed consent, and how can this be effectively conveyed.
